# Evaluation of the effect of afoxalaner with milbemycin 1 oxime in the treatment of rabbits naturally infected with *Psoroptes cuniculi*

**DOI:** 10.1371/journal.pone.0230753

**Published:** 2020-03-27

**Authors:** Camilo Romero Núñez, Ariadna Flores Ortega, Galia Sheinberg Waisburd, Alberto Martin Cordero, Enrique Yarto Jaramillo, Rafael Heredia Cárdenas, Linda G. Bautista Gómez

**Affiliations:** 1 Centro Universitario UAEM Amecameca, Universidad Autónoma del Estado de México, Amecameca de Juárez, Estado de México, México; 2 Centro Veterinario México, Benito Juárez, Cd. de los Deportes, Ciudad de México, México; 3 Veterinaria Dermatología Veterinaria Especializada, Colonia moderna, Guadalajara, Jalisco, México; Beni Suef University, Faculty of Veterinary Medicine, EGYPT

## Abstract

Rabbits (*Oryctolagus cuniculi*) are very popular as pets. However, problems of otitits caused by *Psoroptes cuniculi* are one of the main reasons to visit the veterinarian. Isoxazolines are an alternative treatment to treat this mite, and therefore, an evaluation of the effectiveness of oral afoxalaner with milbemycin oxime in rabbits infected with *P*. *cuniculi* was carried out. Nineteen rabbits, of New Zealand breed, with otitis due to an infection with *P*. *cuniculi*, were treated, whereas six rabbits were left untreated and formed the control group. The ear canals of each individual were examined, through the collection of otic exudate samples with cotton swabs. These were visualized under the microscope to identify the ectoparasite. Each animal was treated with a single oral dose of 2.50 mg / kg of afoxolaner, and 0.50 mg / kg of milbemycin oxime. Clinical signs and lesions associated with the infection, such as the presence of detritus, cerumen and / or scabs, and erythema, were evaluated. After receiving the treatment, all the lesions were classified as: mild, moderate and intense, with a visual analog scale. A week after providing medication, there was a decrease in the lesions of the group treated with Nexgard Spectra®, without further topical or systemic treatment. The decrease was gradual in the treated group and no recurrence was detected of *P*. *cuniculi* infection in both ears. Thus, the administration of a single oral dose of afoxolaner with milbemycin oxime was effective for the treatment of *P*. *cuniculi* infection in rabbits.

## Introduction

The domestic rabbit (*Oryctolagus cuniculi*) has a wide phenotypic diversity of more than 200 breeds worldwide[1]. This is reflected in its multiple commercial uses, such 39 as the production of meat and skin. However, the domestic rabbit is also used as a pet[2], according to the 2006 survey of the American Association of Veterinary Medicine (AVMA), on pet ownership in the US. Currently, rabbits are gaining even more popularity as pets, as it is shown by the increase to 6.1 million pet rabbits, from the 4.8 million documented in the 2001 survey[3]. Therefore, it is necessary to know what are the most suitable treatments for this sort of pets. For rabbits, it is common to present dermatological problems related to the mite *Psoroptes cuniculi* (*P*. *cuniculi*). This parasite is one of the most frequent and causes infection, mainly in the ears[4]. The examination of these patients shows otocariasis with cutaneous inflammation, erythematous pruritic, crustacea, and severe exudative. The Psoroptic mites are found mainly within the pinnae, where scabs are created. Erythema, scabbed lesions in the external ear canals, and pain on palpation are the main clinical findings[5]. In addition, *P*. *cuniculi* can spread to other parts of the host's body, causing generalized pruritus and dermatitis with scabs in the head, neck, ventral abdomen and urogenital area. *P*. *cuiniculi* can infect the owners of infected rabbits because this mite is a zoonotic parasite[6], hence, visiting the veterinarian is mandatory when clinical signs related to mites are present[7]. Several treatments have been tested for scabies caused by *P*. *cuniculi*, e.g., the use of macrocyclic lactones (ML), such as ivermectin, moxidectin, eprinomectin, selamectin, and doramectin[8]. Due to the fact that the mite life cycle is 21 days, the treatments are long and can extend up to a period of 3 to 4 weeks, and require the administration of at least two doses[9]. As an alternative, the use of oral fluralaner, 25 mg / kg dose, is reported as an effective treatment in a single application[10]. (Nexgard Spectra®) is a tablet that contains both afoxalaner and milbemycin oxime constituents, and it is indicated in dogs as a treatment of ectoparasites, but there are no reports on its use in rabbits. Therefore, the objective of this research was to evaluate the effectiveness of afoxalaner with oral milbemycin oxime in rabbits infected with *P*. *cuniculi*.

## Material and methods

### Animals

In this investigation, all the considered animals and procedures followed the recommended practices of laboratory animal care, according to the Mexican Norm (NOM-062-ZOO-1999). The study was approved by the Institutional Care and Use Committee (IACUC) of the University Center Amecameca with register number (201/CUAMECAMECA/UAEMEX/2018).

### Methods of sacrifice

Rabbits were obtained from the experimental farm of the University Center UAEM Amecameca, therefore it was not necessary to sacrifice them. The animals were not treated with analgesics during the experiment, at the end of the experiment it was no longer necessary to administer analgesics due to the recovery of the lesions.

However, during the experiment a visual scale of pain, vital signs, food consumption and behavior was used and in none of the cases were analgeics administered, the animals in the control group at the end of the experiment were treated just like the Nexgard Spectra group®.

Twenty-five rabbits of indistinct genus, with an average age of 1.3±0.29 years, and an average weight of 3.92±0.37, of New Zealand breed, naturally infected with *Psoroptes cuniculi* (*P*. *cuniculi*), were used. The rabbits were in the university zootechnical center, and were kept on a diet based on commercial food for rabbits and water ad libitum, in individual cages. All the rabbits were physically evaluated: the ear canal was examined and the presence of scabs, ceruminous fluid, and inflammation was detected, through a Welch Allyn® veterinary otoscope. Subsequently, swabs were taken to confirm the presence of *P*. *cuniculi* in larval, nymph or adult stages, using the optical microscopy Leica DFC300 FX®. As a previous requirement, they should not have received any type of treatment with avermectins or other drugs used to control external parasites, at least for 60 days prior to the study.

### Treatment

This is the first study on the use of this molecule in rabbits. The dose recommeded for dogs was used because in a previous study, carried out with another isoxazoline (Fluralaner) in rabbits, 25mg/kg was used, just as it is recommended for dogs. These doses were determined with allometric scaling. The animals were divided into two random groups: 19 animals became part of the experimental group, and 6 of the 94 control group. The experimental group was treated with 2.50 mg / kg of afoxolaner, 95 and 0.50 mg / kg of milbemycin oxime (Nexgard Spectra®). The control group 96 received no treatment, and evaluations were made during the days: 1, 7, 14, 21, and 28, after treatment. All treatments and evaluations were performed by the same person. In both groups the food intake and body weight parameters were observed, and a physical examination was carried out in order to determine (heart rate, respiratory rate, and body temperature).

### Sample analysis

During each evaluation day, the ear canals were checked, establishing the presence or absence of *P*. *cuniculi* in any of its stages. This was determined through the presence or absence of parasites, by means of the microscopic examination of the earwax of each ear canal, at 10x objectives, and then at 40x. Additionally, images and videos were obtained, and clinical signs and lesions associated with *P*. *cuniculi* infection were also evaluated through the presence of detritus and / or cerumen, scabs, and erythema. All lesions were classified as mild, moderate and intense, with a visual analogue scale by Sheimberg et al., 2017, (Classification of lesions). Mild: the visual inspection shows the presence of detritus and / or cerumen, and it is possible to see all the ear canal normally observable in the species. Moderate: the visual inspection shows the presence of detritus and cerumen, with a partial visual obstruction (50%), and most of the ear canal normally observed in the species cannot be seen.

### Statistical analysis

A normality test was applied[11] (Shapiro Wilk, 1965), and later the data were analyzed by means of Tukey's Studentized Range Test, to compare the average percentage of lesions per treatment and per week. To carry out the comparison of the amount of parasites in ears, Fisher's exact test was used.

## Results

The result analysis of the degree of ear lesions in the treated group (Table 1) showed that in the first week (pre-treatment) 73% had severe lesions and 100% had some degree of lesions. In the second week (post-treatment), the ears had a higher proportion of minor lesions (71.1%), while severe lesions decreased to zero. By the third week, 92% of the ears of the treated group no longer showed any degree of lesions, and by the fourth week 100% of the ear lesions resolved without further topical or systemic treatment.

**Table 1:** Decrease in lesions per week in the ears of the rabbits naturally infected with *Psoroptes cuniculi*, treated with afoxalaner / milbemycin oxime.

	Week 1	Week 2	Week 3	Week 4
Without lesion	0/38 (0%)	1/38 (2.6%)	35/38 (92.1)	38/38 (100%)
Mild	1/38 (2.6%)	27/38 (71.1%)	3/38 (7.9%)	0/38 (0%)
Moderate	9/38 (23.7%)	10/38 (26.3%)	0/38 (0%)	0/38 (0%)
Severe	28/38 (73.7%)	0/38 (0%)	0/38 (0%)	0/38 (0%)
Total	(100%)	(100%)	(100%)	(100%)

The results presented in this table correspond only to the ears of those rabbits treated with Nexgard Spectra® (n = 38). There were rabbits that presented mild, moderate or severe lesions differently in each ear, and the infections found in these rabbits were both bilateral and unilateral. The ears of the rabbits in the control group did not present changes during the experiment.

Table 2 shows the comparison between the percentages of ear lesion reduction in both groups. In the first pre-treatment week there was no significant difference in the presence of lesions. In the second week the group treated with Nexgard Spectra® presented a decrease in the percentage of injuries, from 86.18% to 30.92%, and 136 when compared to the control group, there was a significant difference (p <0.05). In the third week a significant difference was observed, the group treated showed a decrease in the percentage of injuries (1.97%), contrary to the control group that showed an increase during the second week. In the fourth week there was also a significant difference between groups, in the treated rabbits the lesions resolved, and in the control group they persisted or increased in comparison with the initial measurement.

**Table 2:** Percentage of lesion decrease per week in the ears of those rabbits inoculated with *Psoroptes cuniculi*, treated with afoxalaner / milbemycin oxime, in comparison to the control group.

	Week 1	Week 2	Week 3	Week 4
Nexgard	86.18^a^	30.92^b^	1.97^b^	0^b^
Control group	83.33^a^	83.33^a^	87.50^a^	87.50^a^
CV	27.7	63.3	172.7	186.8
MEE	3.35	3.89	5.49	5.54

^ab^ Averages with a different literal present a significant difference, p <0.05

The positive ear data for *Psoroptes cuniculi* were compared between both groups per week. Table 3 shows the results of the comparison between the number and the percentage of positive ears *to P*. *cunicul*i, analyzing both left and right ears. From the second week onwards, there was a significant difference in the number of positive ears among the rabbits treated with Nexgard Spectra® (0/19 positive ear), and those in the control group (6/6 positive ears); this result was reflected in the decrease in lesions, because each ear improved differently, depending on the degree of the initial lesion.

**Table 3:** Number of positive ears to *Psoroptes cuniculi* per week, among rabbits treated with afoxalaner / milbemycin oxime, in comparison to the control group.

	Week 1	Week 2	Week 3	Week 4
Left ear	Positive (%)	Positive (%)	Positive (%)	Positive (%)
Nexgard n = 19	19 (100)	0 (0)	0 (0)	0 (0)
Control group n = 6	6 (100)	6 (100)	6 (100)	6 (100)
P	1	0.001	0.001	0.001
Right ear				
Nexgard n = 19	19 (100)	0 (0)	0 (0)	0 (0)
Control group n = 6	6 (100)	6 (100)	6 (100)	6 (100)
P	1	0.001	0.001	0.001

Fisher's exact test, P <0.05

## Discussion

The choice of a certain drug for the treatment of *P*. *cuniculi* is an important issue for the veterinarian. Despite the fact that avermectins have been reported as effective against *P*. *cuniculi*, and have been used[12] frequently, some studies on these molecules have revealed the risk of toxicity and problems related to environmental contamination[13]. Besides, the use of isoxazolines is another alternative reported as efficient for the treatment of a variety of mites in pets and exotic animals[14]. There is a case report of 15 New Zealand white rabbits (*Oryctolagus cuniculus*) infected naturally with Psoroptes cuniculi and treated with fluralaner (Bravecto) with favorable results, where no adverse effects were observed[15]. There are no published reports on the pharmacokinetics, safety and efficacy of afoxalaner with milbemycin oxime in rabbits, therefore this would be the first report of a complete remission in rabbits infected naturally with P. cuniculi, treated with a single oral dose of 2.5 mg afoxalaner with 0.50 mg / kg of milbemycin oxime. In previous research regarding this treatment in dogs, vomiting and diarrhea were observed sporadically in all the treated groups[16]. In a previous report, ivermectin combined with a single topical application of fipronil spray proved to be successful in eliminating *P*. *cuniculi* infection. However, the manufacturers of fipronil (Frontline®, Merial) strongly recommend avoiding this product in rabbits due to serious adverse effects. However, the rabbits treated with Nexgard Spectra® in this study did not present any type of adverse reactions, and no clinically relevant changes were observed in food intake, body weight or physical examination parameters (e.g., heart percentage, respiratory percentage, and body temperature). In other studies carried out in dogs, it is described that afoxalaner / milbemycin oxime has a high bioavailability (73.9%), resulting in a rapid and consistent absorption[17]. In regard to rabbits, this information is still unavailable, however, in this study similar results were obtained and are reflected in Table 1, with a decrease in lesions during the week after treatment in the group treated with Nexgard Spectra®, without further topical or systemic treatment.

The above can be compared to the results of the study conducted by Acar *et al*. 2007, and Mellgren *et al*. 2008, in which additional topical and systemic treatments were necessary; this treatment consisted of a subcutaneous injection of ivermectin 400 μg / kg, washes of the ear canal with a topical solution of 0.1% iodine in water, and the administration of gentamicin solution in the auditory canals. In another study, the effectiveness of selamectin treatment and ivermectin was observed with the application of at least two doses, requiring its administration for up to two months. In addition, plasma concentrations of afoxolaner decreased slowly, mainly due to the slow metabolism and excretion of the drug in dogs[18], as it is shown in Table 2. Besides, there was a gradual decrease in the lesions of the group treated with Nexgard Spectra®, contrary to the control group that had an increase. Similarly to a previous study conducted by Sheinberg, *et al*. 2017, on another isoxazoline in rabbits, a residual effect was observed clinically in the treated rabbits without detecting the recurrence of *P*. *cuniculi* infection in both ears (Table 3).

## Conclusion

In conclusion, 2.50 mg / kg of afoxolaner and 0.50 mg / kg of milbemycin oxime gradually reduced the lesions presented by *P*. *cuniculi* in 19 rabbits, without further topical or systemic medication. The treatment was effective in both ears of each individual.

## Supporting information

S1 File (XLSX)
